# Analysis of the potential association between ferroptosis and immune in hepatocellular carcinoma and their relationship with prognosis

**DOI:** 10.3389/fonc.2022.1031156

**Published:** 2023-01-12

**Authors:** Kai Wen, Feng Yang, Lei Hu, Juanyi Shi, Sintim Mui, Weidong Wang, Hao Liao, Huoming Li, Zhiyu Xiao, Yongcong Yan

**Affiliations:** ^1^ Department of Hepatobiliary Surgery, Sun Yat-Sen Memorial Hospital, Sun Yat-Sen University, Guangzhou, China; ^2^ Department of General Surgery, Affiliated Dongguan Hospital, Southern Medical University (Dongguan People’s Hospital), Dongguan, China; ^3^ Department of Pathology, The Seventh Affiliated Hospital, Sun Yat-Sen University, Shenzhen, China

**Keywords:** hepatocellular carcinoma, ferroptosis, immune, immunotherapy, sorafenib, gene signature

## Abstract

**Background:**

The development of targeted therapy and immunotherapy has enriched the treatment of hepatocellular carcinoma (HCC), however, have had poor or no reponse, or even no response. Previous research suggested that ferroptosis and tumor immune microenvironment (TIME) may have a fundamental impact on efficacy during HCC immunotherapy and targeted therapy. Therefore, there is a clinical need to develop a signature that categorizes HCC patients in order to make more accurate clinical decisions.

**Methods:**

Clinical data and gene expression data of HCC patients were obtained from The Cancer Genome Atlas (TCGA) portal and International Cancer Genome Consortium (ICGC) portal. To identify ferroptosis-related immune-related genes (ferroptosis-related IRGs), Pearson correlation analysis was conducted. The ferroptosis-related IRGs prognostic signature (FIPS) was constructed using Univariate Cox and LASSO Cox algorithms. The predictive effectiveness of FIPS was evaluated using Receiver Operating Characteristic (ROC) curves and survivorship curve. The correlation ship between FIPS and TIME was evaluated using single-sample Gene Set Enrichment Analysis (ssGSEA) and CIBERSORT. The relationship between FIPS and immunotherapy responsiveness was evaluated using immunophenoscore. The expression level of 10 ferroptosis-related IRGs in normal liver tissues and HCC tissues was compared using immunohistochemistry. Finally, we established a nomogram (based on FIPS, TNM stage, and age) for clinical application.

**Results:**

The FIPS was established with ten ferroptosis-related IRGs. The high-FIPS subgroup showed a poor clinical prognosis and an obviously higher proportion of HCC patients with advanced TNM stage, high WHO grade and high alpha fetoprotein(AFP) value. Analysis of TIME indicated that patients in the high-FIPS subgroup may be in immunosuppressed state. Meanwhile, we found that ferroptosis may be inhibited in the high-FIPS subgroup and this subgroup may be impervious to immunotherapy and sorafenib.

**Conclusion:**

We constructed a novel potential prognostic signature for HCC patients that predicts overall survival, ferroptosis and immune status, sorafenib sensitivity, and immunotherapy responsiveness.

## Introduction

Primary liver cancer is one of the most common malignant tumors in digestive system. Hepatocellular carcinoma (HCC) is the predominant type (75%-85%) of primary liver cancers with a poor prognosis and high recurrence rate (1). Even among patients who undergo early hepatectomy, more than half of HCC patients relapse within 2 years (2). Sorafenib is the first-line targeted therapy for advanced HCC, but the median survival time after sorafenib treatment is only 12.3 months (3). One clinical research found that the combination of bevacizumab (anti-VEGF antibody) and atezolizumab (anti-PDL1 antibody) improves overall survival (OS) in patients with HCC (4). Moreover, guidelines state that, based on some phase III data, three drug regimens (cabozantinib, regorafenib, and ramucirumab) are approved for the treatment of advanced HCC after progression on sorafenib. In addition, the U.S. FDA approved additional treatment regimens (pembrolizumab and nivolumab and nivolumab combined with ipilimumab) following first-line treatment with sorafenib based on a portion of the Phase Ib/II studies (5–7). However, despite many immunotherapy regimens have been approved, in clinical practice, it has been found that some patients have a poor response to immunotherapy, or even no response. Therefore, there is a clinical need to construct a signature that classifies HCC patients to make more accurate clinical decisions.

Ferroptosis, a newly found pattern of programmed cell death, different than traditional cell apoptosis and necrosis, and results from iron-dependent lipid peroxide accumulation (8).Multiple studies show that ferroptosis is regulated by multiple genes (Ferroptosis-related genes, FRGs), including suppressors of ferroptosis such as Glutathione peroxidase 4 (GPX4) (9) and Solute carrier family 7 member 11 (SLC7A11) (10), and drivers of ferroptosis such as Tumor protein p53 (TP53) (11) and Acyl-CoA synthetase long chain family member 4 (ACSL4) (12). It is noteworthy that one study indicated that CD8^+^ T cells can promote ferroptosis by inhibiting the tumor expression of SLC7A11 (13). Another study noted that CD8^+^ T cells suppress cystine uptake by tumor cells through down-regulation of SLC3A2 and SLC7A11 expression and promote ferroptosis and lipid peroxidation in tumor cells. In addition, that study pointed out that T cell-promoted tumor ferroptosis is an antitumor mechanism; combining this pathway with immunotherapy will offer a novel concept for the treatment of cancer (14). Furthermore, ferroptotic cancer cells could affect antitumor immunity by releasing signals such as oxidized lipid mediators (15). The above studies indicate that ferroptosis is closely related to tumor immunity, but there are few studies on its mechanism.

Sorafenib is the first oral multi-kinase inhibitor approved for the treatment of advanced HCC patients (16). ACSL4 is the driver of ferroptosis, and an *in vitro* study found that the high expression of ACSL4 may increase the sensitivity of HCC cells to sorafenib (17).

As a result, ferroptosis and tumor immune microenvironment (TIME) may have a fundamental impact on efficacy during HCC immunotherapy and sorafenib therapy. In our study, we developed and validated a novel and robust signature using the ferroptosis-related immune-related genes (ferroptosis-related IRGs) classifier for HCC survival. Then we investigated the connection between this signature and ferroptosis status, immune cell infiltration, sorafenib sensitivity and immunotherapy responsiveness in patient with HCC. In addition, we established a nomogram for clinical application.

## Methods and materials

### Data acquisition

The mRNA expression files {Normalized by log_2_[Transcripts Per Kilobase Million (TPM) + 1]} and corresponding clinicopathological data of HCC patients were downloaded from The Cancer Genome Atlas (TCGA)^1^ ^,2^ and the International Cancer Genome Consortium (ICGC)^3^ portal. Exclude patients with missing important clinical information or OS< 30 days to improve the accuracy of our study. Ultimately, we got a training cohort included 343 patients with HCC (from the TCGA) and a validation cohort included 229 patients with HCC (from the ICGC). Then, 227 ferroptosis-related genes were filtrated from previous publications and the FerrDb^4^ database. And 1738 IRGs were screened from the ImmPort Portal^5^. **Table 1** showed the clinical data of each patient in this study.

**Table 1:** Clinicopathological data of each cohort in our study.

		TCGA		ICGC	
		Number	Percentage	Number	Percentage
**Total**		343	100.00%	229	100.00%
**Age**		16-90 (61)		31-89(69)	
	**<median**	165	48.10%	114	49.78%
	**≥median**	178	51.90%	115	50.22%
**Gender**					
	**Male**	233	67.93%	168	73.36%
	**Female**	110	32.07%	61	26.64%
**AFP**					
	**<400**	199	58.02%	-	-
	**≥400**	61	17.78%	-	-
	**NA**	83	24.20%		
**WHO Grade**					
	**I-II**	214	62.39%	-	-
	**III-IV**	124	36.15%	-	-
	**NA**	5	1.46%		
**TNM Stage**					
	**I-II**	238	69.39%	141	61.57%
	**III-IV**	83	24.20%	88	38.43%
	**NA**	22	6.41%	-	-

NA, Not Applicable.

### Establishment of ferroptosis-related IRGs prognostic signature in TCGA cohort

To identify ferroptosis-related IRGs, we first performed Pearson correlation analysis of FRGs and IRGs (with the Pearson *R*<-0.6 or >0.6 and *p<*0.001). Pearson correlation analysis can be used to determine whether the two variables have a linear correlation. Among them, |Pearson R|≤1, the greater the |Pearson R| value, the stronger the correlation between the two variables (18). Univariate Cox regression analysis was performed to filtrate the prognostic ferroptosis-related IRGs. According to the prognostic ferroptosis-related IRGs, ten of them were identified using the LASSO algorithm, and the coefficient of each gene was obtained. Eventually, the formulae for the ferroptosis-related IRGs prognostic signature (FIPS) score calculation was as follows:


$$FIPS\;score=\sum_{i=1}^{n}Coef_{i}*x_{i}$$

Where *x*_i_is the expression is the expression [log_2_ (TPM+1)] of each ferroptosis-related IRG and *Coef*_*i*_ is the coefficient.

Taking the median FIPS score as the cutoff value, HCC patients were classified into high-FIPS and low-FIPS group.

### Functional analysis

Gene Set Enrichment Analysis (GSEA) was used to analysis enrichment of significant pathways. The prognostic ferroptosis-related IRGs and their co-expression FRGs were inputted into the STRING database to form a protein-protein interaction (PPI) network. And Cytoscape (3.8.0) was used for visualization.

Identification of differentially expressed genes (DEGs) between the high-FIPS and low- FIPS subgroups using the R package “limma” (| log2(Fold change). > 1 and adjust p< 0.05). Metascape tool^6^ was used for Gene Ontology (GO) and Kyoto Encyclopedia of Genes and Genomes Pathway (KEGG) analysis.

Gene mutation data of HCC patients in TCGA cohort were obtained from TCGA database and the R package “maftools” was used to analyze the differences in genomic alterations between the high-FIPS and low-FIPS groups.

### Analysis of tumor‐infiltrating immune cells

To determine the characteristics of the immune microenvironment in each HCC patient, single-sample GSEA (ssGSEA) was used to calculate the degree of infiltration of immune cells in each HCC patient and Pearson correlation test was used to analyze the link between FIPS and the degree of infiltration of immune cells and the results are shown in a scatter diagram with the significance threshold set as *p*< 0.05. Moreover, we also used CIBERSORT method to assess the characteristics of the immune microenvironment of HCC patients in TCGA cohort.

### Analysis of the response of immunotherapy

Immunophenoscore (IPS) was constructed based on the expression of MHC molecules, immune checkpoints (ICs), immunsuppressive cells, and effector cells that can accurately predict the response of immune checkpoint inhibitors (ICIs) (19). In our study, we downloaded the IPS values of all HCC patients in TCGA cohort from The Cancer Immunome Atlas (TCIA)^7^ and the difference in IPS values between the low-FIPS and high-FIPS subgroups was compare using Wilcoxon signed-rank test. In addition, we also contrasted the expression of multiple important ICs in the two subgroups using Wilcoxon signed-rank test.

### Analysis of the IC_50_ of sorafenib

Based on the TCGA database, we calculated the half maximal inhibitory concentration (IC_50_) of sorafenib using R package “pRRophetic”. And the Wilcoxon signed-rank test was used to analyze the difference in IC_50_ between the two subgroups. The results are shown in a box plot.

### Analysis of immunohistochemistry staining

With the approval of the ethics committee of Sun Yat-Sen Memorial Hospital, Sun Yat-Sen University, we obtained tumor and adjacent normal tissues of HCC patients from Sun Yat-Sen Memorial Hospital, Sun Yat-Sen University. Then, Protein expression of RORC and PGF in tumor and adjacent normal tissues were detected according to the standard immunohistochemistry staining procedure. Information for antibodies is as described by Proteintech: RORC (13205-1-AP; dilution: 1:600), PGF (10642-1-AP; dilution: 1:400). For the remaining eight prognostic ferroptosis-related IRGs in FIPS, we downloaded the corresponding immunohistochemistry images from the human protein atlas (HPA).

### Statistical analyses

To test the universal applicability of FIPS, log-rank test and Kaplan-Meier curves were used to compare OS between multiple subgroups. Chi-square test was used to compare the distribution of clinical features in the low-FIPS and high-FIPS subgroups (**Table 2**). To prove the independence of FIPS, multivariate and univariate COX regression analyses were conducted in the ICGC and TCGA cohorts.

**Table 2:** Clinicopathological features in two subgroups.

		TCGA			ICGC		
		Low-FIPS	High-FIPS	*p* value	Low-FIPS	High-FIPS	*p* value
**Total case**		172	171		115	114	
**Age**				0.306			0.261
	**<median**	78	87		53	61	
	**≥median**	94	84		62	53	
**Gender**				0.465			0.625
	**Male**	120	113		86	82	
	**Female**	52	58		29	32	
**AFP**				0.176			-
	**<400**	111	88		-	-	
	**≥400**	28	33		-	-	
	**NA**	33	50				
**WHO Grade**				<0.001			-
	**I-II**	126	88		-	-	
	**III-IV**	43	81		-	-	
	**NA**	3	2				
**TNM Stage**				0.001			0.001
	**I-II**	133	105		83	58	
	**III-IV**	29	54		32	56	
	**NA**	10	12				

NA, Not Applicable.

**Figure 1:**
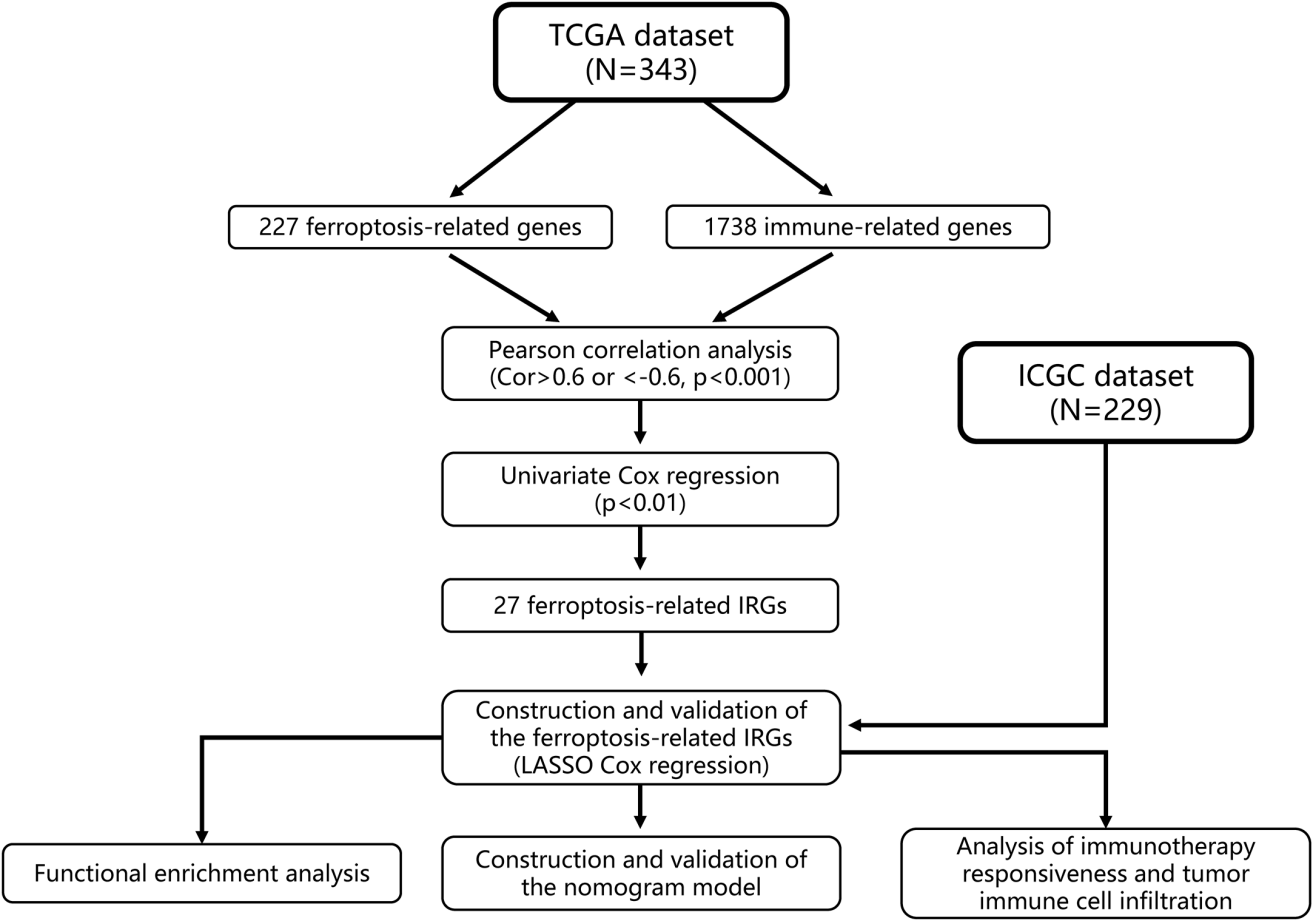
Study flow chart.

We then established a nomogram based on FIPS, TNM stage and age. The prediction accuracy of nomogram was evaluated using the calibration plots and C-index in both two cohorts. The above analysis was performed by package “rms” in R. And we used the R package “timeROC” to plot ROC curves, and the area under the curve (AUC) values were used to show the prognostic capability of the nomogram and other predictors (FIPS, age, TNM stage) for OS (at 1, 2, 3, and 5 years). The flow chart of this study is shown in **Figure 1**. IBM SPSS Statistics 25 and R Programming Language (4.0.0) were used to statistical analysis in this study.

## Results

### Screening ferroptosis-related IRGs in HCC patients

First, we obtained the 227 FRGs from previous publications and the FerrDb database, and 1738 IRGs were downloaded from ImmPort. We extracted the corresponding gene expression matrices from the TCGA dataset. The IRG was defined as a ferroptosis-related IRG when its expression value was correlated with the 227 FRGs (Pearson *R*<-0.6 or >0.6 and *p<*0.001). We ultimately obtained 233 ferroptosis-related IRGs, 27 of which were significantly connected with OS [Identifying by univariate Cox regression analysis (*p<*0.01)]. The outcome of univariate Cox analysis of the 27 ferroptosis-related lRGs is shown in **Table 3**.

**Table 3:** The twenty-seven ferroptosis-related prognostic lRGs.

ferroptosis-related lRGs	HR	HR.95L	HR.95H	pvalue
**BIRC5**	1.618257746	1.278352897	2.048540852	6.29822E-05
**PLXNA1**	1.594039914	1.218569062	2.085202495	0.000667875
**PGF**	1.387135534	1.134284766	1.696350905	0.001436753
**PLAUR**	1.324494915	1.081885108	1.621509314	0.006481143
**IKBKE**	1.303719052	1.078085898	1.576575086	0.00622995
**LPA**	0.861561575	0.775168388	0.957583358	0.005711364
**LECT2**	0.859223934	0.794044541	0.929753599	0.000163567
**AR**	0.838387308	0.747518224	0.94030253	0.002598926
**MASP2**	0.834782374	0.741681807	0.93956951	0.002761419
**TRGC2**	0.826470476	0.718113923	0.951177001	0.00785942
**ACKR2**	0.821200285	0.722126755	0.933866393	0.002672974
**CD8A**	0.82100709	0.709787356	0.949654339	0.007919148
**ITK**	0.81492373	0.701212613	0.947074644	0.007604528
**IL27**	0.811994278	0.709218351	0.929663914	0.002559387
**ADRB2**	0.809658174	0.709126737	0.924441746	0.001799735
**AQP9**	0.804933474	0.705876384	0.917891449	0.001200784
**TMPRSS6**	0.803969476	0.685166116	0.943372567	0.007484104
**ZAP70**	0.775531401	0.650459308	0.924652699	0.004611566
**RORC**	0.769438031	0.663983278	0.891641255	0.000492194
**PROC**	0.749322338	0.60798054	0.923522926	0.006810606
**KNG1**	0.745717101	0.617748969	0.900194128	0.002253632
**KDR**	0.731866159	0.595593569	0.899318098	0.002983581
**RORA**	0.728698964	0.580519142	0.914702275	0.006360066
**RBP5**	0.706549737	0.548838165	0.909580571	0.007031642
**KLKB1**	0.68536195	0.552653813	0.849937142	0.00058022
**RBP7**	0.662561344	0.511430623	0.858352073	0.0018316
**HLA-DRB5**	0.643174204	0.466736823	0.886309022	0.006982544

### Construction of FIPS in the TCGA dataset

**Figure 2:**
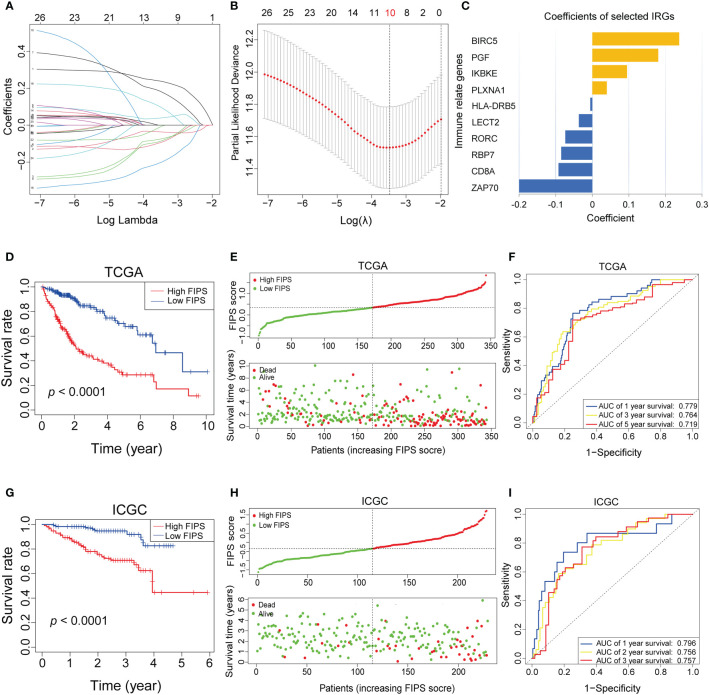
**(A–C)** Least absolute1 shrinkage and selection operator (LASSO) regression was performed, calculating the crucial genes **(A, B)** and coefficients **(C)**. **(D)** Kaplan–Meier curves showed that the high-FIPS subgroup had worse overall survival than the low-FIPH subgroup in The Cancer Genome Atlas (TCGA) cohort. **(E)** Distributions of FIPS scores and survival status of HCC patients in the TCGA cohort. **(F)** Receiver operating characteristic (ROC) curves of FIPS for predicting the 1/3/5-year survival in the TCGA cohort. **(G)** Kaplan–Meier curves showing that the high-FIPS subgroup had worse overall survival than the low-FIPS subgroup in the International Cancer Genome Consortium (ICGC) cohort. **(H)** Distributions of FIPS scores and survival status of HCC patients in the ICGC cohort. **(I)** ROC curves of FIPS for predicting 1/2/3-year survival in the ICGC cohort.

There were 27 prognostic ferroptosis-related IRGs input into the LASSO algorithm and resulting in a 10-gene signature (FIPS; the 10 genes included BIRC5, IKBKE, PGF, PLXNA1, HLA-DRB5, LECT2, RORC, RBP7, CD8A and ZAP70) (**Figures 2A–C**). Calculating FIPS score of all patients according formulae and taking the median FIPS score as the cutoff value, HCC patients were classified into high-FIPS and low-FIPS subgroups. Kaplan–Meier curves indicated that high-FIPS patients had shorter survival times and lower survival rates (**Figure 2D**). **Figure 2E** showed the distributions of FIPS score and survival status. And the ROC curves indicated that FIPS score had excellent predictive capabilities for OS (AUCs at 1, 3, and 5-year reached 0.779, 0.764 and 0.719; **Figure 2F**).

### Validating the FIPS in the ICGC dataset

We used the same formula to calculate FIPS score of HCC patients in the ICGC cohort to validate the ability of FIPS to predict prognosis. We obtained the same results as in the TCGA cohort. The **Figures 2G, H**, showed that, the survival time of patients in the high-FIPS subgroup was significantly shorter than that in the low-FIPS subgroup (*p*< 0.001). In addition, the ROC curves (**Figure 2I**) also showed that FIPS score had excellent predictive capabilities for prognosis in the ICGC cohort (The AUCs of 1, 2, and 3-year reached 0.796, 0.757 and 0.756). All these results indicate that FIPS is a reliable prognostic signature with a strong ability to predict OS in HCC patients.

### Prognostic analysis of the ten ferroptosis-related IRGs

**Figure 3:**
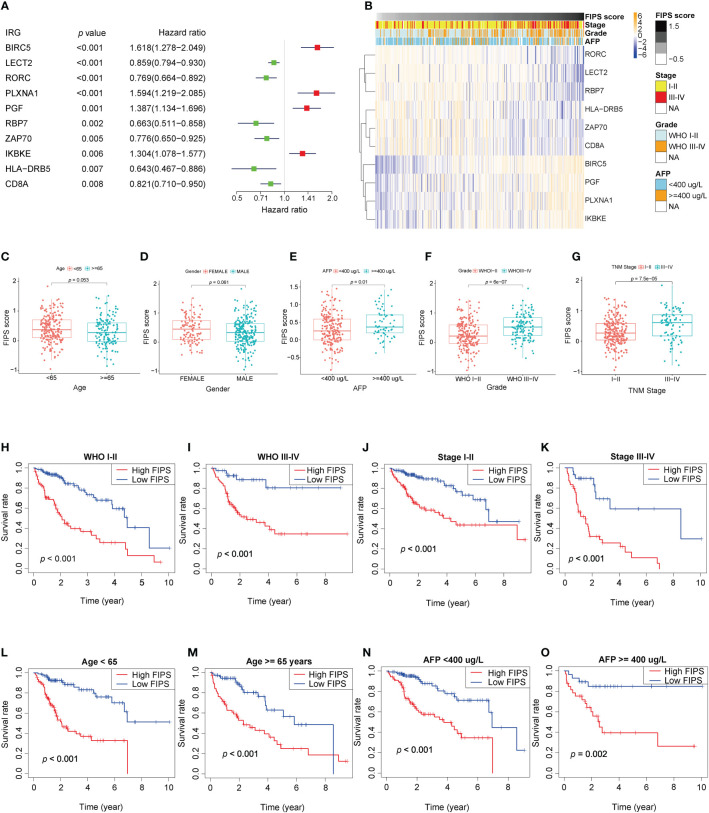
**(A)** Forest plot of the prognostic ability of the ten ferroptosis-related IRGs included in the prognostic signature. **(B)** Heatmap of the associations between the expression levels of the ten ferroptosis-related IRGs and clinicopathological features in the TCGA cohort. **(C–G)** Patients with different clinicopathological features (including AFP value, WHO grade, TNM stage, but not age and gender) had different levels of FIPS scores. **(H–O)** The FIPS retained its prognostic value in multiple subgroups of HCC patients (including patients with WHO grade III-IV or I-II, TNM stage III-IV or I-II, age<65 or ≥ 65 years and AFP< 400 or ≥ 400 ug/L).

We used Univariate Cox regression to analysis the relationship between 10 ferroptosis-related IRGs and prognosis, and the results are shown in a forest plot. LECT2, RORC, RBP7, ZAP70, HLA−DRB5, and CD8A were protective factors with a hazard ratio (HR)<1, whereas BIRC5, PLXNA1, PGF, and IKBKE were risk factors with HR >1 in patients with HCC (**Figure 3A**). And the heatmap shows that the expression levels of 10 ferroptosis-related lRGs had significant differences/were significantly different between the high- and low-FIPS subgroups. The expression levels of BIRC5, PLXNA1, PGF, and IKBKE were elevated with increasing FIPS score. In contrast, LECT2, RORC, RBP7, ZAP70, HLA−DRB5, and CD8A expression levels were reduced with increasing FIPS score. Moreover, the expression levels of ten ferroptosis-related lRGs were associated with the clinical characteristics of HCC, such as AFP, TNM stage, and WHO grade (**Figure 3B**).

### Stratification analysis of the FIPS

We did stratification analyses to explore the connection between FIPS score and clinicopathological features of HCC patients. The box plot shows that HCC patients with TNM Stage III-IV, AFP ≥ 400ug/L, and WHO grade III-IV had higher FIPS score but FIPS score was not related to gender and age (**Figures 3C–G**). To comprehensively evaluate the prognostic power of FIPS, multiple subgroups analyses were used to determine the predictive capabilities of FIPS in different subgroups. The survival curves showed that FIPS had good prognostic ability in multiple subgroups [Including TNM stage (I-II and III-IV), WHO grade (I-II and III-IV), age (≥65 and<65 years) and AFP (≥400ug/L and<400ug/L) subgroups] (**Figures 3H–O**). These results indicated that FIPS is a widely applicable prognostic signature for HCC patients.

### Independent prognostic value of FIPS

**Figure 4:**
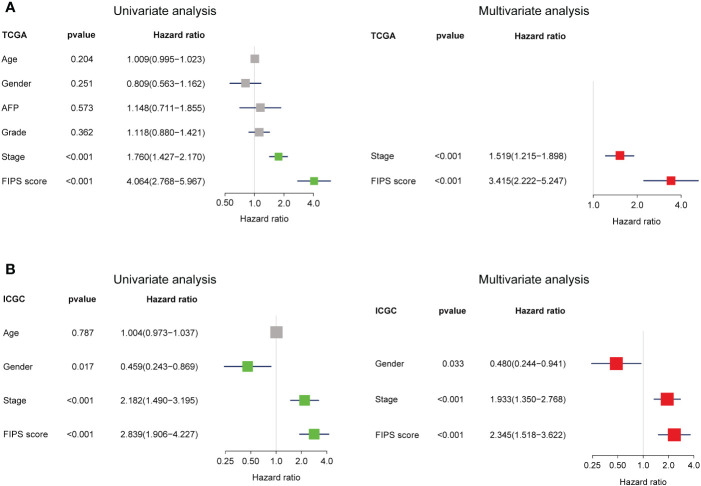
**(A, B)** Univariate and multivariate analyses revealed that FIPS score was an independent prognostic predictor in the TCGA and ICGC cohorts.

To verify the independence of FIPS, multivariate and univariate Cox regression analyses were conducted in both the ICGC database and TCGA database. In TCGA cohort, according to univariate Cox regression analysis, FIPS score (HR = 4.064, 95% confidence interval (CI) = 2.768−5.967, *p<*0.001; **Figure 4A**) was significantly associated with OS. Multivariate analysis by Cox regression showed that, after correcting for other confounding factors, FIPS score (HR = 3.415, 95% CI = 2.222−5.247, *p<*0.001; **Figure 4A**) was an independent risk factor. We also validated this result in the ICGC cohort (**Figure 4B**). The results described above showed that FIPS is a prognostic factor independent of other clinical features.

### Development and validation of prognostic nomogram

**Figure 5:**
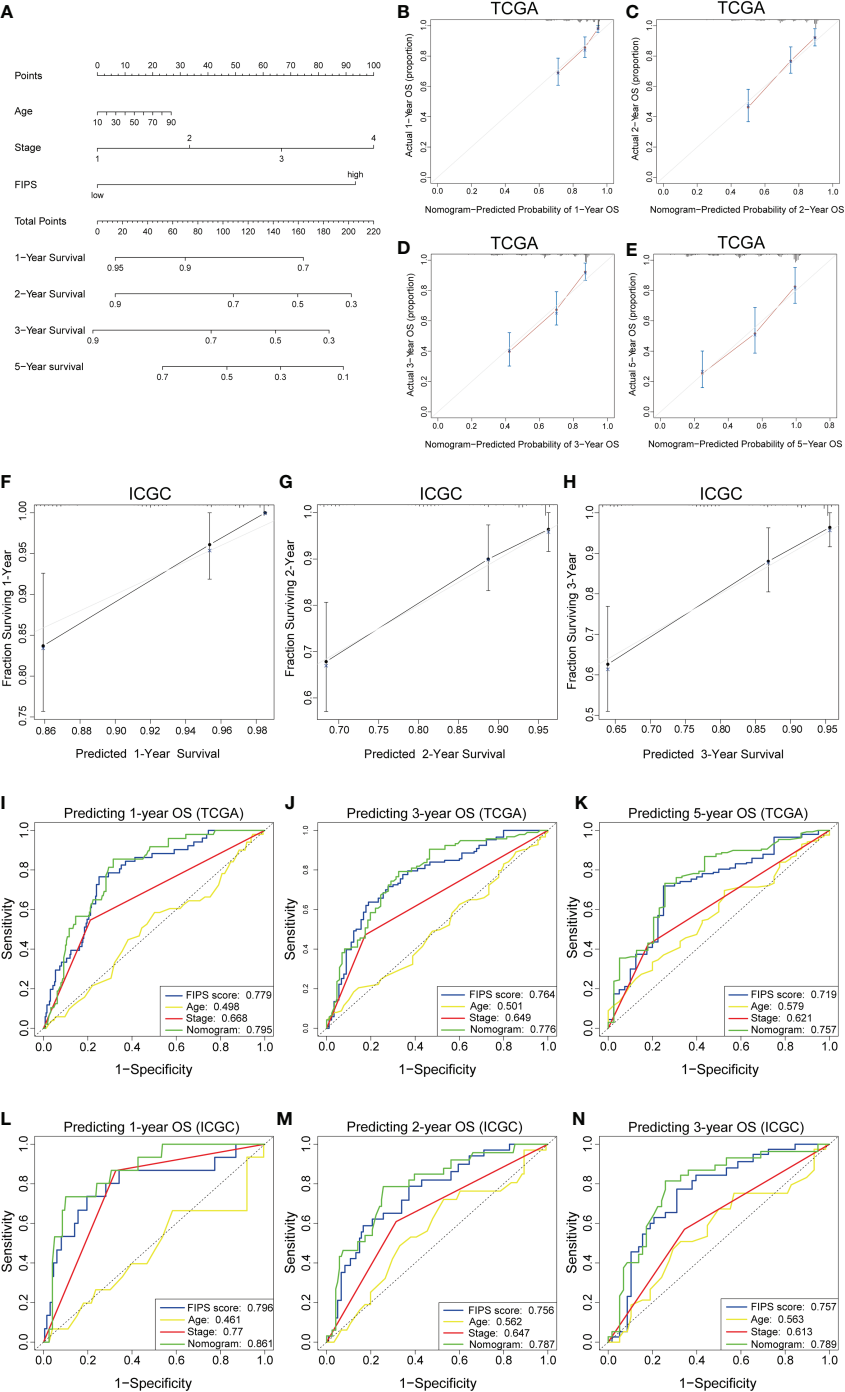
**(A)** Nomogram based on FIPS, age and TNM stage. **(B-E)** Calibration plots of the nomogram for predicting the probability of OS at 1, 2, 3, and 5 years in the TCGA cohort. **(F-H)** Calibration plots of the nomogram for predicting the probability of OS at 1, 2, and 3 years in the ICGC cohort. **(I–K)** Time-dependent receiver operating characteristic (ROC) curves for the nomogram, FIPS score, age and TNM stage in the TCGA cohort (for predicting 1, 3, and 5-year OS). **(L–N)** ROC curves for the nomogram, FIPS score, age and TNM stage in the ICGC cohort (for predicting 1, 2, and 3-year OS).

To further improve the prediction accuracy of FIPS, we established a nomogram based on FIPS, TNM stage and age in the TCGA cohort (**Figure 5A**). Calibration plots indicated almost perfect concordance between predictive and actual survival both in TCGA (**Figures 5B–E**) and ICGC cohorts (**Figures 5F–H**). In addition, the ROC curves indicated that the nomogram showed excellence prognostic predictive ability (In TCGA, 1, 3, and 5-year AUC = 0.795, 0.776, and 0.757; in ICGC, 1, 2, and 3-year AUC = 0.861, 0.787, and 0.789; **Figures 5I–N**). Moreover, the results of C-index showed that the nomogram had a strong and reliable prognostic capability (C-index in TCGA cohort: 0.729 and ICGC cohort: 0.787). These results showed that the nomogram had a strong and reliable prognostic capability for HCC patients.

### Functional analysis

**Figure 6:**
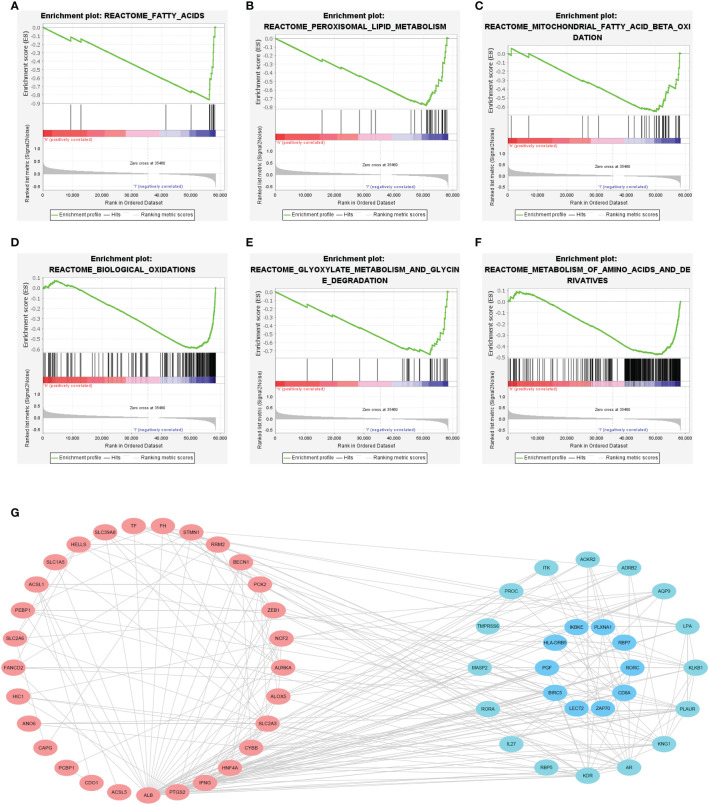
**(A–F)** Gene set enrichment analysis (GSEA) of the high-FIPS and low-FIPS subgroups in the TCGA cohorts. **(G)** A protein-protein interaction (PPI) network suggesting the relationship between ferroptosis-relate genes (FRGs) and immune-relate genes (IRGs).

The GSEA showed that several ferroptosis-related biological processes were significantly enriched in the low-FIPS subgroup, including fatty acid metabolism, peroxisomal lipid metabolism, biological oxidations, and glycine degradation **(Figures 6A-F)**. These findings indicate that ferroptosis might be inhibited in the high-FIPS subgroup.

The PPI network of 27 IRGs and their co-expressed FRGs showed that there was a strong correlation between the IRGs and FRGs (**Figure 6G**).

The results of GO and KEGG analysis showed that these DEGs were enriched in the following pathways: mitotic cell cycle, chromosome segregation, Metapathway biotransformation Phase I and II, microtubule cytoskeleton organization involved in mitosis, and androgen metabolic process (**Supplementary Figure S1**).

### Analysis of tumor‐infiltrating immune cells and response to immunotherapy

**Figure 7:**
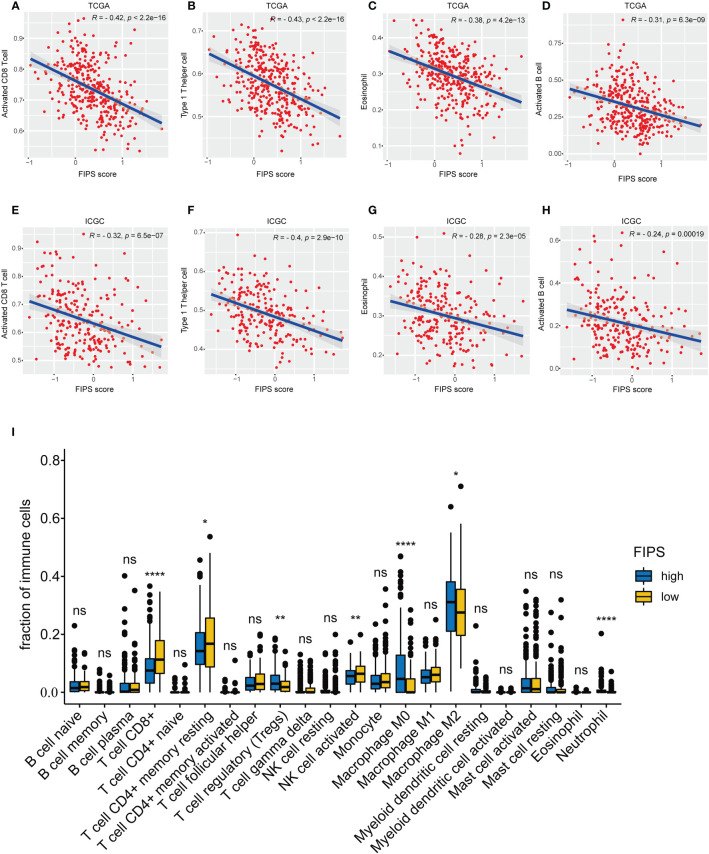
**(A–H)** single-sample Gene Set Enrichment Analysis (ssGSEA) and correlation analysis of the FIPS score and the immune enrichment scores of immune categories in the TCGA and ICGC cohorts. **(I)** Comparison between the fractions of immune cells in the high- and low-FIPS subgroups of the TCGA cohort *via* the CIBERSORT method. *p< 0.05; **p< 0.01; ****p< 0.0001.

First, we investigated the connection between FIPS and tumor‐infiltrating immune cells by ssGSEA in both two cohorts. The results showed that the Activated CD8^+^ T cells, Activated B cells, Type 1 T helper cells (Th1 cells) and eosinophils were significantly negatively correlated with FIPS scores (**Figures 7A–H**). The detailed correlation analysis of 28 immune cells with FIPS score is shown in **Supplementary Figure S2C**. CIBERSORT analysis in TCGA cohort further indicated that the CD8^+^ T cells, resting memory CD4^+^ T cells, and activated natural killer cells significantly infiltrated the low-FIPS subgroup while the neutrophils, M0 macrophage, M2 macrophage and regulatory T cells (Tregs) significantly infiltrated the high-FIPS subgroup (**Figure 7I**). Notably, M2 macrophages are the predominant tumor-associated macrophages (TAMs) (20). Considering TAMs, Tregs and Tumor-associated Neutrophils (TANs) are well-researched immunosuppressive cells (21). We speculate that the high-FIPS subgroup may have an immunosuppressive status.

**Figure 8:**
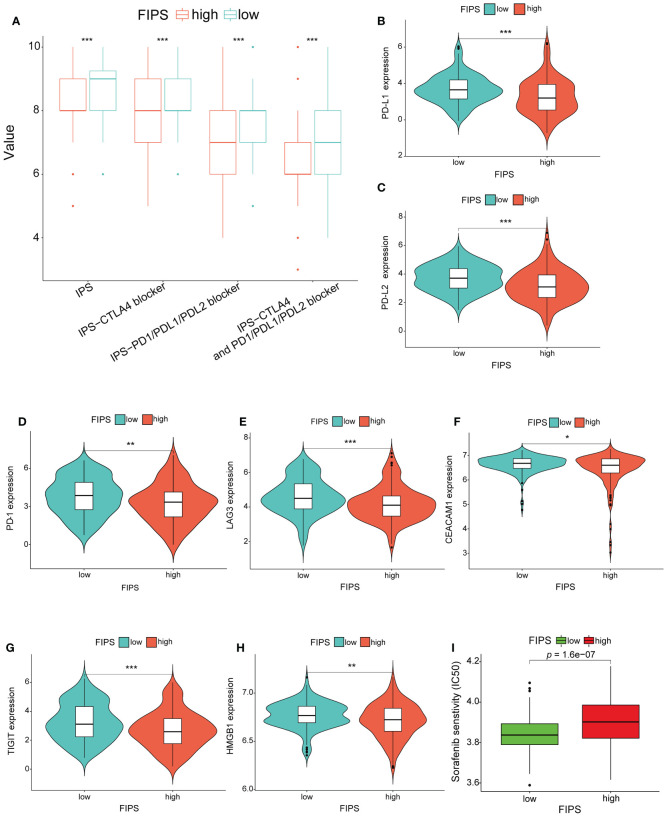
**(A)** Comparison between the immunophenoscore (IPS) value in the high- and low-FIPS subgroups in the TCGA cohort. **(B-H)** Comparison between the expression of several prominent immune checkpoints in the high- and low-FIPS subgroups of the TCGA cohort. **(I)** Comparison between the half maximal inhibitory concentration (IC_50_) of sorafenib in the high- and low-FIPS subgroups of the TCGA cohort. *p < 0.05; **p < 0.01; ***p < 0.001; ****p < 0.0001.

In order to further analyze the relationship between FIPS and immunotherapy, we downloaded the IPS value of all patients in TCGA cohort from TCIA and then compared the differences in IPS values between low-FIPS and high-FIPS subgroups. The results show that the value of IPS-CTLA4- and PD-L1/PD1/PD-L2 blocker, IPS-PD-L1/PD1/PD-L2 blocker, IPS-CTLA4 blocker and IPS were higher in the low-FIPS subgroup, which suggests that the response to immunotherapy in the low-FIPS subgroup might be better than that in the high-FIPS subgroup (**Figure 8A**). We further analyzed the relationship between the expression of 10 ferroptosis-related IRGs and IPS values, and the results showed that the expression of RBP7, LECT2, ZAP70, RORC, CD8A and HLA-DRB5 were significantly positively correlated with IPS values, while the expression of BICR5 and PLXNA1 were significantly negatively correlated with IPS values. The expression of PGF and IKBKE had no significant correlation with IPS values. This suggests that upregulation of RBP7, LECT2, ZAP70, RORC, CD8A, and HLA-DRB5 may be beneficial to immunotherapeutic responses, whereas BICR5 and PLXNA1 are not (**Supplementary Figure S2A**). For sorafenib, we analyzed the relationship between the expression of 10 ferroptosis-related IRGs and IC50 of sorafenib. The result showed that the expressions of PGF, ZAP70, CD8A, IKBKE, BICR5 and PLXNA1 were positively correlated with IC50 of sorafenib, while the expressions of HLA-DRB5, RORC, LECT2 and RBP7 were significantly negatively correlated with IC50 of sorafenib. These results suggest that high expression of PGF, ZAP70, CD8A, IKBKE, BICR5 and PLXNA1 may be associated with sorafenib resistance (**Supplementary Figure S2B**). In addition, we analyzed the expression differences in several critical ICs in the low-FIPS and high-FIPS subgroups. And the results are presented in **Figures 8B–H**, showing that the expression levels of PD-L2, PD-1, PD-L1, LAG3, CEACAM1, TIGIT, and HMGB1 were significantly up-regulation in the low-FIPS subgroup. These results indicated that the low-FIPS subgroup might have a better immunotherapy responsiveness than the high-FIPS subgroup.

### Analysis of the relationship between FIPS and sorafenib sensitivity

To analyze the efficacy of sorafenib in the low-FIPS and high-FIPS subgroups, we used the R package “pRRophetic” to calculate the IC_50_ of sorafenib in the two subgroups, respectively, in the TCGA database. And the results of Wilcoxon signed-rank test indicated that, compared with the high-FIPS subgroup, patients in the low-FIPS subgroup were more sensitive to sorafenib (**Figure 8I**).

### Analysis of gene mutation

We used R package “maftools” to process the gene mutation data of HCC patients in TCGA cohort. We found that, in the high-FIPS group, the top five genes with mutation frequency were TP53 (42%), TTN (24%), MUC16 (15%), CTNNB1 (16%), and PCLO (10%) while those in the low-FIPS group were CTNNB1 (33%), TTN (20%), MUC16 (15%), TP53 (14%), and ALB (12%). Further analysis showed that TP53, ANK1, CELSR3, SPECC1 and SORCS3 with mutation frequencies were higher in the high-FIPS group, while NLRP12, CSMD2, SF3B1, and NAV3 with mutation frequencies were higher in the low-FIPS group (p< 0.05) (**Supplementary Figures S3B-D**).

### Analysis of immunohistochemistry staining

**Figure 9:**
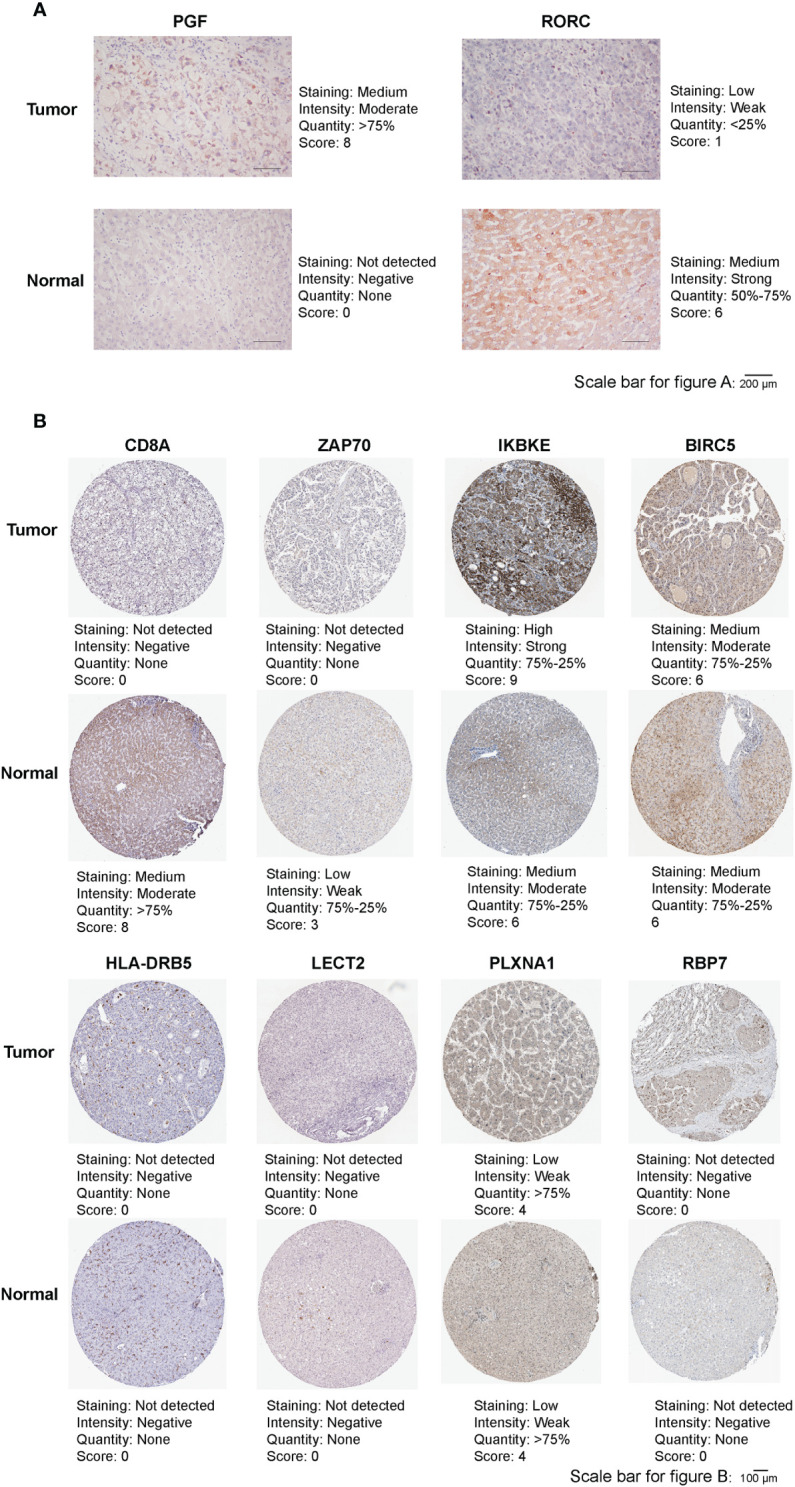
**(A)** Representative immunohistochemistry images of RORC and PGF in HCC tissues and adjacent normal tissues. **(B)** Representative immunohistochemistry images of CD8A, ZAP70, IKBKE, BIRC5, HLA-DRB5, LECT2, PLXNA1 and RBP7 in HCC tissues and normal tissues.

Immunohistochemistry was Conducted to detect protein expression of RORC and PGF in our HCC tissue and adjacent normal liver tissue. As **Figure 9A** showed that RORC protein expression was higher in adjacent normal tissues and PGF protein expression was higher in HCC tissues. In addition, the **Figure 9B** showed that, IKBKE protein expression was significantly increased in HCC tissues, whereas CD8A and ZAP70 protein expression were increased in normal liver tissues. For BIRC5, HLA-DRB5, LECT2, PLXNA1 and RBP7, no differences were found in their protein expression between HCC tissues and normal liver tissues.

## Discussion

Although the treatment and diagnosis of HCC have improved, the mortality and morbidity in HCC are still high. The advent of targeted therapy and immunotherapy has significantly improved outcomes for cancer patients, however, it has been found that some patients have poor response to immunotherapy, or even no response. Distinct from surgical treatments, decisions on targeted therapy and immunotherapy often depend on the molecular phenotype or “signature” of tumor cells (22–24). In this study, 343 patients with HCC from a TCGA dataset and 229 from an ICGC dataset were studied to construction the ferroptosis-related IRGs prognostic signature. In the TCGA datasets 10 ferroptosis-related IRGs were used to construct FIPS for predicting prognostic in HCC patients. The high-FIPS subgroup had a poor clinical prognosis and obvious higher proportion of HCC patients with advanced TNM stage, high WHO grade, and high AFP value. analysis of TIME and IPS value also indicated that patients in the high-FIPS group may be in immunosuppressed and immunotherapy unresponsive status. In addition, we found that ferroptosis might be inhibited in the high-FIPS subgroup. Finally, we established a nomogram incorporating FIPS, TNM stage, and age that showed an excellent prognostic capability in both two cohorts.

Among the 10 genes in the FIPS that we constructed, BIRC5 was found to be upregulated in HCC and could promote tumor proliferation by inducing CDK4 to release p21 (25). Currently, several BIRC5 inhibitors are available for the treatment of HCC (26, 27). Some vivo studies indicate that high expression of LECT2 in HCC cells can promote sinusoid capillarization, inhibit portal angiogenesis, and promote liver fibrosis (28). Results from another study indicated that LECT2 is a crucial role in liver tumorigenesis as its absence alters the tumor phenotype and the tumor microenvironment suggested that LECT2 is a promising immunotherapeutic option for HCC (29). MiR-134 could down-regulate PLXNA1 to block the MAPK signaling pathway, and miR-134 overexpression or silencing of PLXNA1 can promote cell apoptosis, delay cell cycling, and inhibit cell proliferation, migration, and invasion (30). IKBKE is a central role in both the occurrence and progression of KRAS-induced pancreatic transformation (31). Notably, we found that IKBKE was also a strong risk factor for HCC. CD8A was found to be predominantly expressed on the surface of cytotoxic T lymphocytes and mediates effective cell-cell interactions within the immune system (32). Consistent with this, in this study, high-FIPS patients were found to be immunosuppressed with low CD8A expression. Several studies have pointed out that HLA-DRB5 plays an important role in Alzheimer disease and multiple sclerosis (33, 34), while the role of HLA-DRB5 in tumors has not been reported. This study indicated that low HLA-DRB5 expression is related to poor outcome in HCC. RBP7 was closely associated with colon cancer invasion and epithelial-mesenchymal transformation, and ectopic expression of RBP7 enhanced invasion and metastasis of colon cancer (35). Interestingly, in our study, the up-regulated expression of RBP7 was related with better prognosis in HCC patients, suggesting that RBP7 may exert distinct biological functions in HCC and deserves further exploration. ZAP70 plays an important role in lymphocyte activation and T-cell development. And ZAP70 mutations could cause severe immunodeficiency disease (36). However, the role of ZAP70 in HCC has not been clarified. RORC is required for IL-17A production from Th-17 cells, which plays a crucial role in the pathogenesis of several inflammatory and autoimmune diseases (37). As the member of the VEGF family, PGF can bind to VEGFR1 to promote tumor angiogenesis and growth, and inhibition of PGF expression can reduce the metastasis and growth of a variety of tumors (38–40). In line with this, our study found that RORC is a positive prognostic factor associated with better OS in HCC, while PGF is a risk prognostic factor associated with poor OS in HCC. On the other hand, functional analysis shows that these IRGs are strongly associated with ferroptosis, the interactions between them are yet to be found out.

Several studies have shown that TIME is a key role in immunotherapy responsiveness and liver cancer progression. For example, the extent of infiltration by CD8^+^ T cells can affect the efficacy of pembrolizumab, and patients with more infiltration are more responsive to pembrolizumab than those with less infiltration (41). And several other studies have shown that increased eosinophils are associated with improved prognosis in solid tumors (42, 43). Th1 cells can coordinate cytotoxic T lymphocytes and NK cells to exert anti-tumor effects by secreting multiple cytokines (IFN-γ, TNF-α and IL-2) (44). Yutaka et al. indicate that infiltration of B cell is an independent positive prognostic factor in HCC (45). Here, we found that Activated B cells, Activated CD8^+^ T cells, Th1 cells and eosinophils were significantly negatively correlated with FIPS scores, suggesting that the poor prognosis and Immunosuppressive status of patients in the high-FIPS subgroup may be related to this. Notably, we found that Tregs, M2 macrophages and neutrophils were high-infiltrated in the high-FIPS subgroup. Tregs and tumor-associated neutrophils (TANs) are well-researched immunosuppressive cells, and Tregs exert immunosuppressive effects by inhibiting dendritic cells and effector T cells through multiple pathways (46–48). TANs could recruit macrophages and Tregs into HCC, thereby promoting the growth and metastasis of HCC, and contributing to sorafenib resistance (21). In addition, reports in the literature indicate that M2 macrophages are the predominant TAMs which could promote tumor progression and inhibit anti-tumor immunity by expressing chemokines and cytokines (20, 21). Taken together, patients in the high-FIPS group may be in immunosuppressed state.

The advent of immunotherapy has raised new hope for patients with HCC (49). Currently, many immunotherapy regimens have been approved, but only a proportion of patients are able to benefit from immunotherapy in clinical practice. The IPS is a good predictor of the response to ICIs (19). Here, we have found that the value of IPS-CTLA4- and PD-L1/PD1/PD-L2 blocker, IPS-PD-L1/PD1/PD-L2 blocker, IPS-CTLA4 blocker and IPS were significantly higher in the low-FIPS subgroup, which suggests that the response to immunotherapy in the low-FIPS subgroup might be better than that in the high-FIPS subgroup. Moreover, we found that expression of several important ICs was significantly up-regulated in the low-FIPS subgroup, such as PD-L1, PD-1, PD-L2, LAG3, CEACAM1, TIGIT, and HMGB1. This further illustrates that the low-FIPS subgroup might have a better response to immunotherapy than the high-FIPS subgroup.

A previous study used 60 FRGs to build a model to predict OS in HCC patients, and the AUCs at 1, 2, and 3 years (TCGA database) were 0.8, 0.69, and 0.668; in the ICGC database, the AUCs at 1, 2, and 3 years were 0.68, 0.69, and 0.718 (50). In addition, there are two studies that combined FRGs and IRGs to predict the prognosis in HCC patients and the 1, 3, and 5-year AUCs of “CIFI” were 0.69, 0.7, and 0.73 in the TCGA database (51, 52). Different from our study, FRGs and IRGs were included together in the model in these two studies. In our study, we first used Pearson correlation analysis to filter ferroptosis-related IRGs and then build the FIPS based on ferroptosis-related IRGs. In addition, there are some studies only considered the single factor of ferroptosis, and the predictive power of the constructed prognostic model was also limited, as its AUC values generally ranged from 0.66 to 0.8, and the AUC values at 1, 3, and 5 years differed greatly (53–56). Compared with these, our model had superior ability to predict OS in HCC patients. And the predictive power of our nomogram (based on FIPS) for OS (In the TCGA database 1, 3, and 5-year AUC = 0.795, 0.776, and 0.757 and in the ICGC database 1, 2, and 3-year AUC = 0.861, 0.787, and 0.789) was strong too. Furthermore, for immunotherapy, we analyzed the IPS values of HCC patients, making our model more reliable to predict immunotherapy efficacy.

In summary, we have established a novel signature based on ferroptosis-related IRGs that can accurately predict prognostic in patients with HCC. And a FIPS-based nomogram has been established for clinical application. Furthermore, FIPS was associated with immune cell infiltration, ferroptosis status, sorafenib sensitivity and response to immunotherapy. Therefore, this study provides a novel method of predicting the response of HCC to targeted therapy and immunotherapy, which may provide clinically valuable information in targeted therapy and immunotherapy of HCC. However, there are some limitations of our study. More independent validation cohorts are needed to validate this model. And more basic research is needed to explore the mechanism by which the 10 ferroptosis-related IRGs we screened play a role between iron death and immunity. Our study can provide clues to the further study of the interaction between ferroptosis and immunity.

## Data availability statement

The original contributions presented in the study are included in the article/**Supplementary Material**. Further inquiries can be directed to the corresponding authors.

## Ethics statement

The studies involving human participants were reviewed and approved by the ethics committees of Sun Yat-Sen Memorial Hospital, Sun Yat-Sen University. Written informed consent to participate in this study was provided by the participants’ legal guardian/next of kin. Written informed consent was obtained from the individual(s), and minor(s)’ legal guardian/next of kin, for the publication of any potentially identifiable images or data included in this article.

## Author contributions

ZX and YY constructed this study. KW, FY and LH performed the data analysis, figures plotted, and writing. JS, SM and HL did the Immunohistochemistry staining. WW and HML were responsible for the data acquisition and critical reading of the manuscript. All authors read and approved the final manuscript.

## Footnotes

1
https://portal.gdc.cancer.gov/.

2
https://xenabrowser.net/.

3
https://dcc.icgc.org/projects.

4
https://www.zhounan.org/ferrdb.

5
https://www.immport.org.

6
www.metascape.org .

7
https://tcia.at/patients.
