# Supplementary material for: Analysis of the potential association between ferroptosis and immune in hepatocellular carcinoma and their relationship with prognosis
Source: Front Oncol. 2023 Jan 12;12:1031156. doi: 10.3389/fonc.2022.1031156 (PMC9910086; doi:10.3389/fonc.2022.1031156)
Supplement: Supplementary file 1 [file Image_1.pdf]

Supplementary Figures

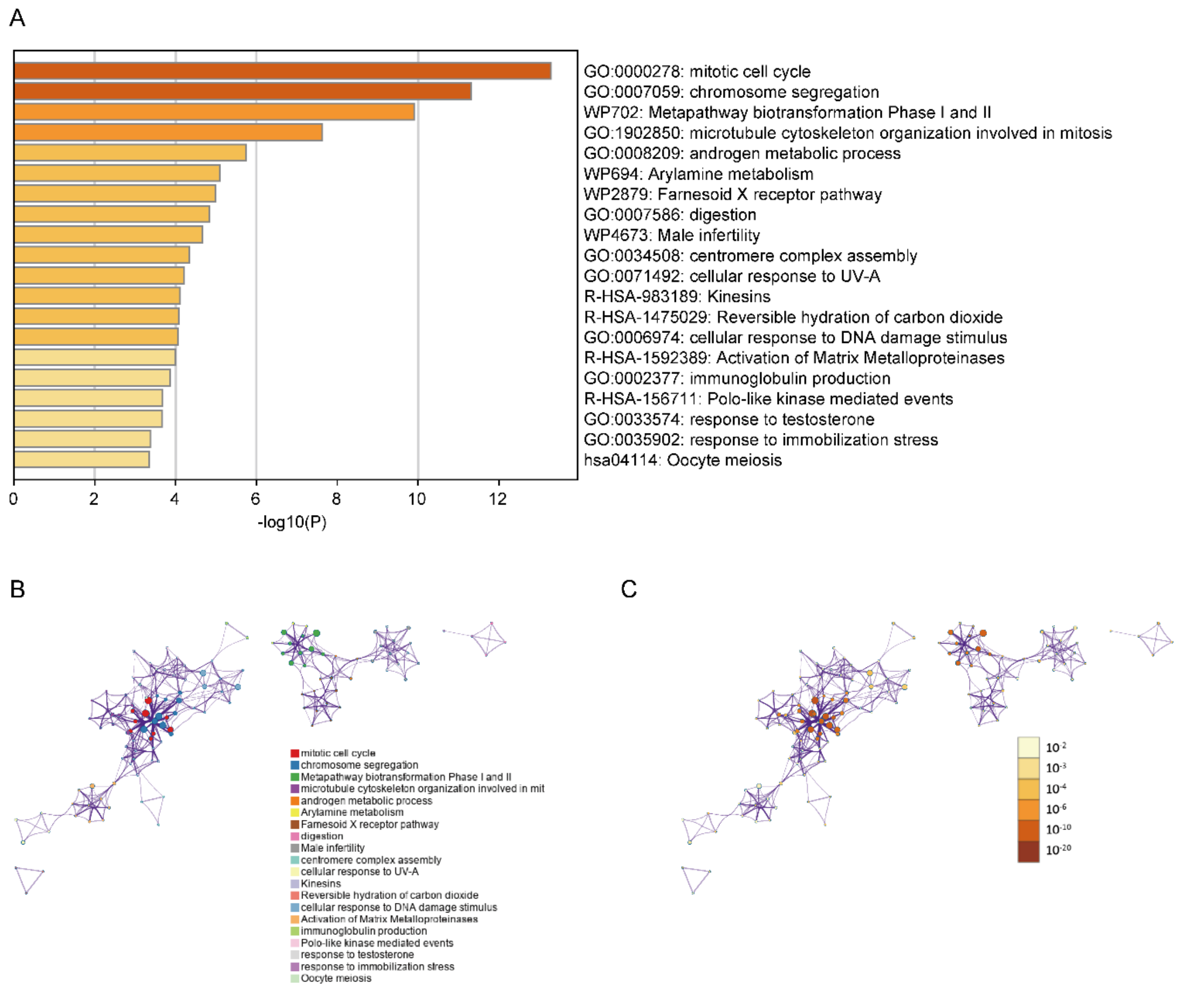

Supplementary Figure 1 | (A) Heatmap of enriched terms across the inputted gene list, colored according to p-value. Network of enriched terms colored according to **(B)** cluster ID (nodes with the same cluster ID are typically close to each other) and **(C)** p-value (terms with more genes tend to have higher p-values).

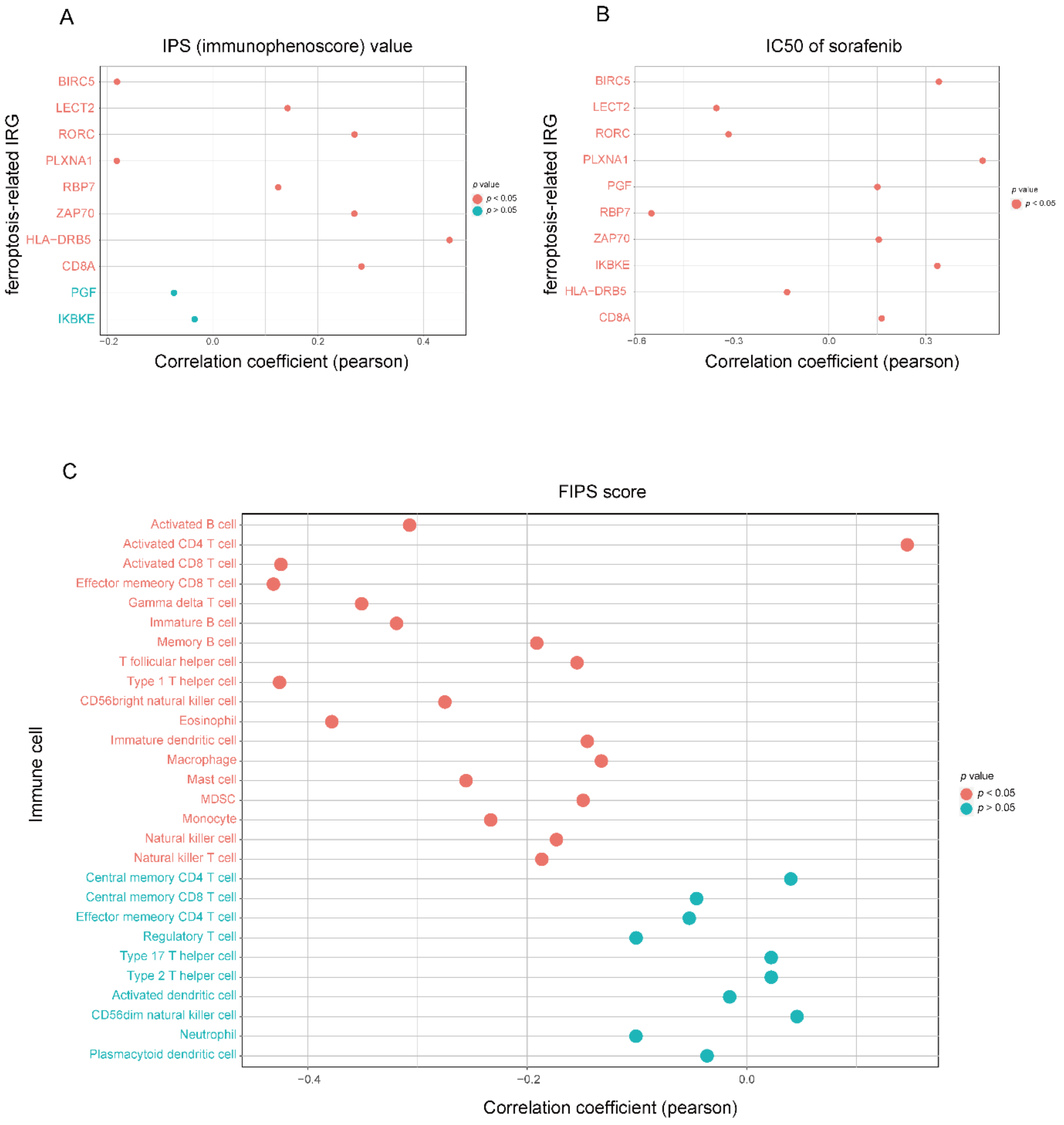

Supplementary Figure 2 | (A) Correlation analysis of the 10 ferroptosis-related IRGs and the IPS (immunophenoscore) value in the TCGA cohorts. **(B)** Correlation analysis of the 10 ferroptosis-related IRGs and the IC50 of sorafenib in the TCGA cohorts. **(C)** single-sample Gene Set Enrichment Analysis (ssGSEA) and correlation analysis of the FIPS score and the immune enrichment scores of immune categories in the TCGA cohort.

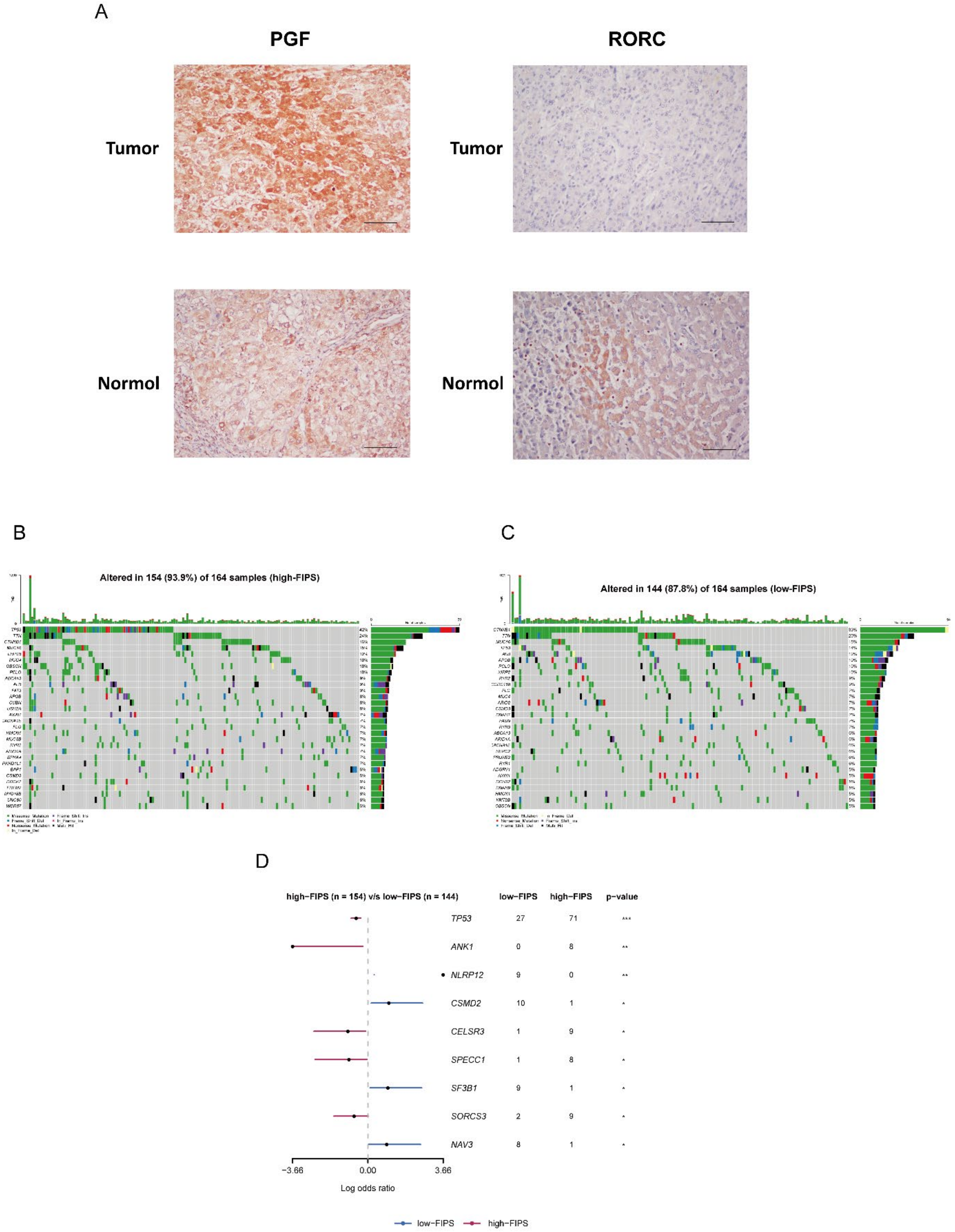

Supplementary Figure 3 | (A) Representative immunohistochemistry images of RORC and PGF in HCC tissues and adjacent normal tissues. **(B-C)** Oncoplots of the mutated genes in the **(B)** high-FIPS and **(C)** low-FIPS groups of the TCGA cohort. **(D)** Forest plot of the differentially mutated genes between the high- and low-FIPS groups.
